# Addressing the implementation challenge of the global biodiversity framework

**DOI:** 10.1007/s10531-020-02009-2

**Published:** 2020-07-01

**Authors:** Sui C. Phang, Pierre Failler, Peter Bridgewater

**Affiliations:** 1 Centre for Blue Governance, School of Business and Law, University of Portsmouth, Portsmouth, PO1 2UP UK; 2 University of Canberra, 11 Kirinari St, Bruce, ACT 2617 Australia; 3 Copernicus Institute of Sustainable Development, Utrecht University, Princetonlaan 8a, Utrecht, The Netherlands

**Keywords:** Convention on biological diversity, Conservation, Policy, Governance, Sustainable development

## Abstract

A Global Biodiversity Framework (GBF) is under discussion for the period 2021–2030, which will replace the “Aichi Targets” adopted by the Convention on Biological Diversity (CBD) in 2010. Given the limited success in meeting most of the Aichi Targets, this new framework must adopt a different approach. A key challenge the GBF must address is its implementation at national scales. Four ways this implementation challenge can be addressed include:

- The framework must move away from numerical targets to pursue positive trends in biodiversity, through adopting a “vectors of change” approach;
- The framework should be structured to focus on ecosystems and processes;
- The framework should synergise more extensively with existing biodiversity-relevant global agreements to maximise leverage and reduce overlap of resource use;
- The framework must adopt a much stronger theory of change than is in the current GBF Draft, to serve as the roadmap governments can use in upscaling their implementation of biodiversity conservation, sustainable use and benefit sharing.

Finally, the GBF must become a “learning framework”, committed to facilitating and enabling governments to each meet their specific biodiversity challenges, while sharing back experiences with the global community, leading ultimately to realising the 2050 CBD vision of people living in harmony with nature.

## Introduction

After a decade of efforts to halt biodiversity loss through the 20 Aichi Targets of the Convention of Biological Diversity (CBD), the situation remains very mixed (IPBES 2019). Overall success in meeting targets has been low and is not uniform by region nor by target. For instance, in Africa, none of the 50 countries convention parties are on course to meet Targets 3, 4, 12 or 20 at all by 2020, and even the most on-track Targets (16 & 17) are only expected to be achieved by 24% of these countries (IPBES 2018). Such an uneven achievement of targets translates to an imbalance in biodiversity conservation, sustainable use, and benefit sharing—the three objectives of the CBD. This situation in Africa is widely representative of the negative trends for global biodiversity in the Anthropocene (IPBES 2019).

The challenge of implementing global biodiversity policies is crucial and must be addressed in the new Global Biodiversity Framework (GBF) slated to replace the Aichi Targets. Given limited success of two decades of CBD target-setting policies, starting from the 2010 Targets set in 2002, adopting another set of numerical biodiversity targets would seem best avoided. Numerical targets, even under a unified single metric (e.g. Rounsevell et al. 2020), distract and divert resources from effective implementation. Instead, a successful GBF must be a framework from which policies are developed and implemented urgently and effectively at local, national, and regional scales. It must overcome the difficulty of accommodating multiple and complex concerns of all stakeholders from environmental, societal, economic and political domains and meet needs of people and biodiversity. The Intergovernmental Science-Policy Platform on Biodiversity and Ecosystem Services (IPBES) Global Assessment (IPBES 2019) calls for transformative approaches to biodiversity governance—here we propose four promising ways the GBF could be transformative for people and the rest of biodiversity.

## Move away from numerical targets to pursue positive trends in biodiversity

The GBF must move from numeric-based targets and instead adopt ‘vectors of change’ as its approach for better biodiversity conservation, sustainable use and benefit sharing. In the released *Zero Draft for a Post-2020 Global Biodiversity Framework*, numeric-based targets for overall biodiversity loss are included (i.e. Section 10 of the Zero Draft), for example, the percentage reduction in the species under extinction threat and increase in species abundance. To calculate these percentages requires robust biodiversity threat assessments and poses substantial implementation challenges for member states with insufficient capacity. The same challenges also apply for gene and ecosystem metrics used in the Zero Draft. Indeed, while metrics to measure the state of biodiversity exist and some are even well established (Mace et al. 2018), without the reliable ability to assess biodiversity in the first instance in many regions of the world, it serves as a ‘paper target’.

Setting biodiversity ambition in the GBF to a “vectors of change” approach describing a better situation for biodiversity (for example see Bull et al. 2020) can maintain an ambitious and desirable objective while also simplifying implementation. The 2030 and 2050 goals in the GBF, focusing on biodiversity status **(**defined by species health, human use and equitable sharing of resources) can be readily adapted from numerical targets to ‘vectors of change’. CBD Parties have the opportunity to define country-level ambition (Maron et al. 2020) that reflect their needs and capacities. At the global level, use of narratives encapsulating biodiversity as the CBD defines it—hierarchical linkage of genes, species and ecosystems—is the best way forward. Through the GBF (via e.g. the Global Environment Facility) the global community must commit to ensure all CBD Parties have appropriate capacity (i.e. expertise, technology, facilities etc.). Ownership of a vector of change narrative encourages buy-in by both government and civil society and will incentivise policy implementation.

Embracing ecological novelty as one vector of change can assist a range of changes in implementing nature conservation (Bridgewater and Hemming 2020; Heger et al. 2020). In the Zero Draft, “recovery of natural ecosystems” appears as one of the main foci for CBD parties towards 2050; however, what “natural ecosystems” means in the Anthropocene remains an open question (Heger et al. 2020). Here, again, setting appropriate narratives, rather than numerical targets, is an approach more likely to yield positive results by 2030.

## Structured to focus on ecosystems and processes rather than activities

The GBF must focus on supporting the pursuit of synergistic activities among stakeholders, themes (e.g. conservation, sustainable use and sharing) and systems (e.g. mountains, coasts, deep sea etc.). This will be critical for achieving the CBD 2050 target of “living in harmony with nature”. Use of predetermined activities in the Zero Draft of the GBF, e.g. protected areas (Section D, a2), is not always conducive for promoting collaboration among human communities with different interests. This is evidenced by Aichi Targets 6 “Sustainable Fisheries” & 11 “Protected Areas”. Indeed, Aichi Target 11 explicitly describes the implementation of more protected areas “to achieve the long-term conservation of nature with associated ecosystem services”, which in the marine context includes fisheries. In practice, however, more protected areas is not always in the interests of biodiversity users (De Santo 2013). Considerable debate still exists how protected areas should be implemented even after a decade of Aichi Targets (Visconti et al. 2019; Woodley et al. 2019).

Encouraging synergies among and providing choices for stakeholders in the GBF can happen through regenerating the CBD Ecosystem Approach (i.e. ensuring the policy scale is that of the ecosystem). For example, a focus on marine systems, rather than separate targets on sustainable fisheries and marine protected areas, will encourage more effective conservation and sustainable use of marine biodiversity. Mobile marine protected areas (Maxwell et al. 2020) can be expanded to include parameters encompassing fisher needs and other CBD objectives as well as traditional protected area objectives. Promoting collective action by fisheries for conservation has precedence, with as extreme example being the role of the International Whaling Commission through its global moratorium on commercial whaling. This effective intervention halted commercial whaling, albeit addressing a self-inflected threat, and led to increases in most whale species populations, including the highly-endangered blue whale *(Balaenoptera musculus*)*.*

## Synergise with existing biodiversity-relevant global agreements

The GBF must be aligned with other biodiversity-related policies and initiatives to maximise leverage for positive behavioural (transformational) change. The intention for the GBF to contribute to, and synergise with, the UN Sustainable Development Goals is both positive and opportune given their overlapping and complementary objectives in biodiversity conservation and use. Promoting mutually beneficial interactions with biodiversity-relevant policies in other global initiatives will help achieve CBD objectives—and the important advances in bringing Indigenous and traditional knowledge to the table must continue and be expanded.

For example, in the marine realm, policies relevant to the GBF include the Biodiversity Beyond National Jurisdiction of UN Convention on the Law of the Sea, International Whaling Commission, and the FAO Code of Conduct for Responsible Fisheries. Indeed, policies do not have to be limited to marine limits and can span biodiversity (CITES, CMS), land–water (Ramsar Convention on Wetlands), cultural (World Heritage Convention) and the other Rio Conventions (UNCCD, UNFCCC).

## A stronger theory of change

The GBF draft adopts a Theory of Change approach that clarifies a pathway to generate the desired change or impact. This is different from the “set and forget” approach that characterised the targets adopted in 2002 and 2010. However, the Theory of Change included in the Zero Draft is too vague in its presentation when clarity, specifically in the steps to achieve action, are needed. Ensuring the necessary conditions and actions are present is critical in determining the ultimate effectiveness of biodiversity actions (Amano et al. 2018).

**Fig. 1:**
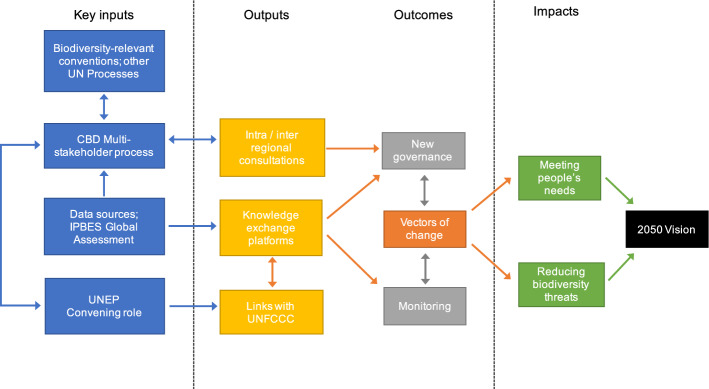
Expanding the Theory of Change presented in the Zero Draft of the Global Biodiversity Framework to identify critical stakeholders and information flows can facilitate implementation at the national level. This theory of change illustrates some of the necessary links to achieve the 2050 vision, or impact, of the CBD

The GBF needs a more elaborated theory of change for effective implementation. The theory of change should identify specific actors and their responsibilities, becoming a map member states can use when designing and implementing actions where they count, i.e. at national level and local scales (Fig. 1). Indeed, the GBF in current format mentions implementing, including stakeholders and the role of the Subsidiary Body on Implementation. However, the attention given to implementation is disproportionately small compared to that given to defining the new biodiversity targets. Defining key narrative-based ‘implementation goals’ are vital to reflect the importance of implementation, which remains a critical challenge.

## Making the GBF and CBD work

In the next decade, the GBF must facilitate creation—and implementation—of biodiversity policies that account for a member state’s physical, ecological, social, cultural and economic dimensions. The challenge for globally defined policies is the need for more and better biodiversity science. And the policies need a clear and effective implementation framework. The global community, through the GBF, must enable member states to build capacity for implementing global biodiversity policies for their specific needs within their specific capacities. In this way the GBF can become a “learning framework” that adapts readily and speedily to change, while maintaining line of sight to the key objective of people living in harmony with nature.

Additional time available, due to the Covid-19 induced postponement of subsidiary body for the CBD on science matters and the next round of GBF discussions, give a prime opportunity to recast and rework successful implementation of the CBD, through the GBF. Greater understanding that implementation is only effective at national level should drive policies and decisions and help in the global transformation of policy and practice, through better governance for biodiversity. Capacity building, knowledge transfer and resource distribution can be delivered effectively only at national level, with regional support where needed. On a promising note, the GBF zero draft and subsequent discussions reflect movement away from adherence to targets and tentatively addresses some of the limitations of previous policies—but more is needed in this direction.

As urgency to finalise the GBF mounts, and as the world, in working through the current global pandemic, ponders how to understand and manage the links between human health and biodiversity health, CBD parties must make their convention finally work effectively. Ultimately, the aim for the new framework, and work programme for the CBD, must be that biodiversity is better conserved, better used and better shared, reaching the impact inherent in the 2050 vision.
